# Cold Water Immersion Does Not Enhance Recovery and Performance After High‐Intensity Interval Dorsiflexion Exercise

**DOI:** 10.1111/sms.70061

**Published:** 2025-05-02

**Authors:** Andrew J. Richards, Rohin Malekzadeh, Mohamed E. Elghobashy, Robert Laham, Geoffrey A. Power, Michael T. Paris, Arthur J. Cheng

**Affiliations:** ^1^ Muscle Health Research Centre, School of Kinesiology and Health Science, Faculty of Health York University Toronto Ontario Canada; ^2^ Department of Medicine University of Toronto Toronto Ontario Canada; ^3^ Department of Human Health and Nutritional Sciences, College of Biological Sciences University of Guelph Guelph Ontario Canada

**Keywords:** cold‐water immersion, exercise performance, exercise recovery, high‐intensity interval exercise, neuromuscular fatigue, prolonged low‐frequency force depression, recovery interventions, skeletal muscle fatigue

## Abstract

Cold‐water immersion (CWI) has become a widely adopted method for post‐exercise recovery. However, its effectiveness in restoring neuromuscular function remains inconclusive. This study examined the impact of CWI on recovery following high‐intensity interval exercise (HIIE). Twelve young, recreationally active individuals (10 males, 2 females) participated in a randomized crossover study. Each session included six sets of 30‐s all‐out isokinetic concentric contractions of the ankle dorsiflexor and plantar flexor muscles, followed by 10 min of room temperature rest (RT) or CWI at 10°C. Neuromuscular function and intramuscular temperature were evaluated periodically over 24 h, as well as next‐day fatigue resistance of the dorsiflexors. In both conditions, maximal voluntary contraction torque remained impaired for up to 3 h without significant changes in voluntary activation (*p* > 0.05). Electrically stimulated torque showed no difference in the fatigue‐induced decline or recovery of 10 Hz torque, which also remained impaired for up to 3 h. However, 50 Hz torque recovered within 1 h following RT, whereas it remained slightly reduced for up to 3 h following CWI. The 10:50 Hz torque ratio showed immediate recovery with CWI, whereas RT recovery was delayed for up to 1 h. Notably, the ratio was significantly lower with RT at 0‐, 0.5‐, and 1‐h post‐intervention. Despite these differences, HIIE performance during a repeat bout conducted 24 h later remained similar. In conclusion, 10 min of CWI at 10°C does not enhance post‐exercise recovery or next‐day exercise performance following HIIE.

## 1 Introduction

Cold‐water immersion (CWI) is a widely used post‐exercise recovery intervention due to its assumed benefits in enhancing the recovery of neuromuscular and metabolic function [1, 2, 3, 4]. The application of CWI is proposed to aid in the recovery of exercise performance by attenuating several exercise‐induced responses such as inflammation and perceived muscle soreness and/or pain via muscle cooling [4, 5, 6] and the hydrostatic effects of CWI restricting local blood circulation and fluid shifts [1, 3, 7, 8, 9, 10, 11]. However, the effectiveness of CWI on affecting muscle function directly and its associated mechanisms remain elusive, especially following high‐intensity exercise [5, 8]. A fundamental function of skeletal muscle during exercise is to contract and produce force, and potential alterations in skeletal muscle function may occur irrespective of observed biomarker changes in inflammatory markers or perceptions of soreness/pain, or vice versa.

One phenomenon that underlies the slow recovery of skeletal muscle function following fatiguing high‐intensity exercise is prolonged low‐frequency force depression (PLFFD) [12]. During the post‐exercise recovery period after intense exercise, PLFFD is observed as a reduction in evoked low‐frequency muscle contractile force that can take hours or even days to fully recover, whereas evoked high‐frequency force (i.e., maximal strength) recovers within minutes in the post‐exercise recovery period [12]. Given that most everyday movements, including sports activities, involve submaximal contractions, the implications of PLFFD are that the depression in submaximal force following fatiguing exercise contributes extensively to the perceived sensation of muscle weakness in the post‐exercise recovery period.

Previously, it has been shown that just one bout of 30‐s all‐out cycling exercise is sufficient to reduce low‐frequency force after the initial bout [13], with repeated cycling sprints causing marked PLFFD [14]. The proposed mechanism causing PLFFD following high‐intensity interval exercise (HIIE) is increased reactive oxygen and nitrogen species (RONS) generation causing decreased sarcoplasmic reticulum (SR) calcium (Ca^2+^) release as well as decreased myofibrillar Ca^2+^ sensitivity [12]. Treatments to combat PLFFD have been proposed, one of which is CWI [12]. It has been suggested that CWI might be able to limit ROS/RONS generation and damage [4, 15] by reducing intramuscular temperature [16] and therefore potentially accelerating the recovery of PLFFD. It has been previously shown that skeletal muscle temperatures above 32°C are associated with greater RONS production [16]. Therefore, rapidly reducing exercise‐induced increases in intramuscular temperature using CWI might be a viable post‐exercise strategy to accelerate muscle recovery and restore force production. However, it is not known if CWI can mitigate PLFFD symptoms after HIIE.

Thus, the aim of this study was to investigate the effects of CWI on the post‐exercise recovery of neuromuscular function following acute HIIE performed in the lower leg. To distinguish the localized effects of CWI from the systemic effects on muscle function [5], CWI in the current study was only applied to the exercised lower leg. We assessed the post‐exercise recovery of neuromuscular function in the first 3 h immediately following CWI to determine whether CWI mitigates the acute development of PLFFD, which could be relevant to sports involving multiple training sessions or competitions in a day (e.g., swimming and track and field sports). Furthermore, next‐day HIIE performed 24 h following the initial bout was evaluated to mimic sports where repeated competitions are performed on consecutive days (e.g., qualification rounds in the Olympics and in professional sports leagues) whereby athletes employ CWI as an immediate post‐exercise recovery modality to improve next‐day performance. It was hypothesized that CWI will be beneficial in accelerating the acute recovery (≤ 3 h) of submaximal force generation, therefore enhancing the reversal of PLFFD. Additionally, it was hypothesized that CWI will have no effect on neuromuscular function at later time points of recovery (i.e., 24 h) or next‐day HIIE performance as it is proposed that the acute effects and physiological changes seen with cooling will have returned to baseline within the first few hours following either recovery intervention the prior day [17], thus not influencing later time points of recovery and subsequent HIIE performance (i.e., 24 h).

## 2 Materials and Methods

### 2.1 Participants

Twelve healthy participants (10 M, 2 F) were recruited to participate in this randomized crossover study. The participants were 23.3 ± 3.1 y of age with an average height of 176.6 ± 8.1 cm and a mass of 84.1 ± 13.5 kg. Participants were recreationally active and not participating in organized sport at the time of testing. Participants were free of acute or chronic disease affecting their neuromuscular performance. Participants were told to refrain from caffeine consumption on the day of testing and to avoid strenuous exercise 24 h before their laboratory visit. All participants gave informed, written consent, and the study was approved by the local ethics committee on human research (York University Human Participants Ethics Review Committee) and conforms to the Helsinki statement.

### 2.2 Experimental Overview

**FIGURE 1:**
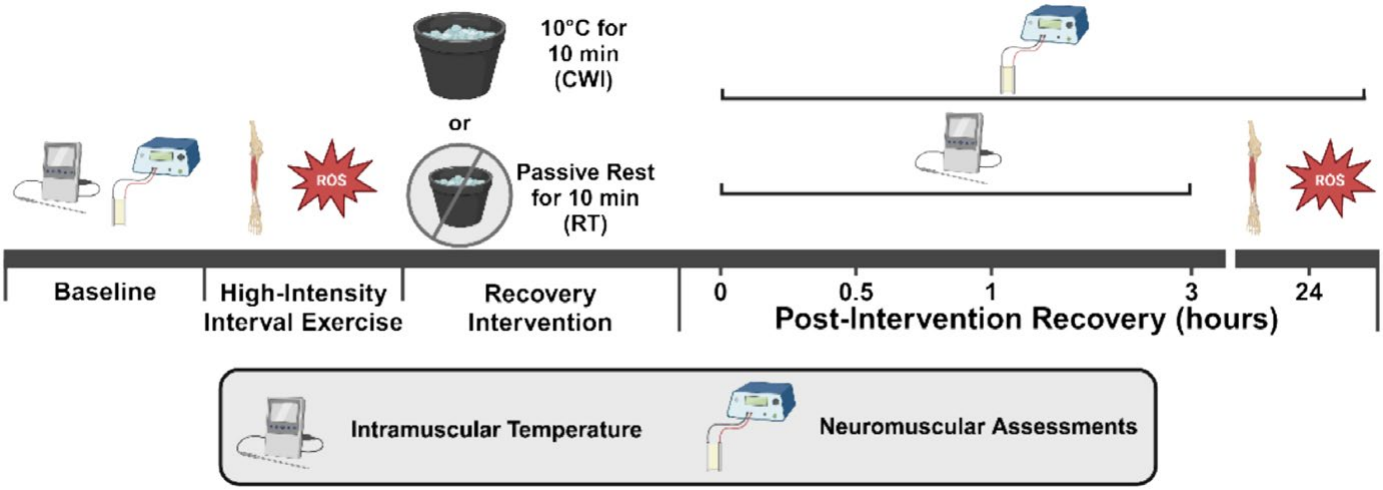
Experimental protocol. Study schematic representing an outline of the experimental procedures.

Experiments consisted of five to six laboratory visits in which neuromuscular function of the ankle dorsiflexors was assessed. The first visit was a familiarization trial for participants to gain an understanding of the experimental protocol and habituate themselves with the maximal effort contractions and the electrical stimulations. If necessary, a second familiarization trial was performed to ensure the participant understood the procedures and could achieve > 90% of voluntary activation (%VA) assessed via a modified interpolated twitch technique (described below). At the start of each experimental session, baseline neuromuscular assessments of the ankle dorsiflexors and intramuscular temperature measures of the tibialis anterior muscle were recorded (Figure 1). Thereafter, participants performed high‐intensity interval concentric dorsiflexion and plantarflexion exercise in the lower left leg. The tibialis anterior muscle was selected based on its size, whereby our CWI protocol, specified below, could be performed as quickly as possible post‐exercise and would be able to drastically reduce intramuscular temperature compared to a larger muscle. Immediately following the fatiguing exercise, and in a randomized order, the lower left leg was placed in either CWI at 10°C for 10 min or kept at room temperature (RT) for 10 min (Figure 1). The immersion temperature of 10°C and duration of 10 min was selected as it is one of the most commonly used immersion protocols within the literature [7, 18, 19, 20] and fits within the range at which CWI is suggested to be most beneficial and tolerable by individuals [1, 2, 9, 20, 21]. Furthermore, we chose to only cool the lower left leg as regardless of whether we did sub‐immersion up to the waist or full immersion up to the neck, similar intramuscular temperatures would be seen in the tibialis anterior muscle and therefore isolate the effects of localized cooling on muscle function irrespective of systemic changes. During the post‐exercise recovery period, neuromuscular assessments were performed at 0 h, 0.5 h, 1 h, 3 h, and 24 h later (the 24 h time was laboratory visits 3 or 4 and 5 or 6 depending on whether a second familiarization session was needed) (Figure 1). Assessments at earlier timepoints (i.e., 0 h, 0.5 h, 1 h, and 3 h) provided information on the acute recovery of neuromuscular function following exercise and CWI, whereby this time period is where PLFFD also recovers at the fastest rate [12]. Furthermore, 24 h after the initial bout of exercise, a second HIIE bout was performed in the previously exercised leg to determine the next‐day recovery of muscle fatigue resistance (i.e., total number of contractions, peak velocity, and peak torque achieved per contraction across sets) (Figure 1). Subsequent experimental sessions were similar, with the only difference being the recovery intervention (i.e., either CWI or RT), which took place within the following 2 weeks to allow for adequate rest and recovery.

### 2.3 Experimental Set‐Up

A HUMAC NORM isokinetic dynamometer (CSMi Medical Solutions, Stoughton, MA, USA) was used throughout the study to record joint torque (N·m), angular velocity (°/s), and joint position (°) values for neuromuscular assessments and exercise performance. Participants were seated with their left hip at 110° of flexion and knee at 120° of extension. Extraneous movement of the torso was restricted with a 4‐point seatbelt harness. The left foot was fixed to a dorsiflexor dynamometer foot pedal with a nonelastic tightening strap positioned over the ankle and two others strapped at the mid‐portion of the foot. The axis of rotation of the isokinetic dynamometer was aligned with the participant's left lateral malleolus. The maximal ankle dorsiflexion angle was set to 30° of plantar flexion and 10° of dorsiflexion (neutral position 0°), allowing for a 40° joint range of motion during the concentric dorsiflexion and plantarflexion contractions during exercise. During the neuromuscular assessments, the ankle joint was fixed at 30° of plantar flexion, as pilot testing data indicated that peak isometric dorsiflexion torque was achieved at that joint angle, which is consistent with previous literature [22].

For the neuromuscular assessments, a clinical bar electrode (Model E.SB010, Digitimer North America, Fort Lauderdale, FL, USA) was used to stimulate the peroneal nerve to evoke isometric dorsiflexor contractions. The peroneal nerve was found by locating the fibular head and moving both inferiorly and posteriorly to determine the optimal position to maximally activate the dorsiflexor muscles using electrical stimulation. Single pulses (single 200‐μs square wave pulses, 400 V) from a constant current stimulator (Model DS7R; Digitimer, Welwyn Garden City, UK) were delivered to the peroneal nerve to evoke dorsiflexion twitches. The maximal current intensity was determined by 5 mA increases until a plateau in the recorded twitch force was observed.

### 2.4 Baseline Voluntary and Involuntary Neuromuscular Assessment

To determine the %VA current when using the modified interpolated twitch technique, the maximal current was increased further by 30% (65.2 ± 19.2 mA), which was subsequently used for all evoked twitch and doublet (100 Hz) stimulations. When employing the interpolated twitch technique, participants were verbally encouraged to contract as forcefully as possible for ~3–5 s during the maximal voluntary isometric contraction upon which a superimposed 100 Hz doublet was evoked, which was immediately followed by a potentiated resting doublet 1–2 s following full relaxation. Visual feedback on a computer monitor was provided to the participants of their dorsiflexion torque generated during these maximal efforts. The %VA was calculated using the following equation: %VA = (1—interpolated doublet torque/potentiated resting doublet torque) × 100 [23]. To assess peripheral fatigue as well as PLFFD, the peak torque achieved during the 10 Hz and 50 Hz tetanic stimulations was obtained, respectively, and a ratio of the 10–50 Hz torque was calculated [24]. Both the 10 Hz and 50 Hz were evoked for a duration of 1.5 s, respectively. The current used for the low‐frequency (10 Hz) and high‐frequency (50 Hz) involuntary isometric tetanic stimulations was based on the same current used for the 100 Hz doublets, as performed previously to ensure supramaximal stimulation of electrically evoked contractions [25]. The low‐ to high‐frequency torque ratio (i.e., 10:50 Hz ratio) is used to infer exercise‐induced changes in intramuscular Ca^2+^ regulation where greater impairments to force are seen at low versus high frequencies due to decreased SR Ca^2+^ release and decreased myofibrillar Ca^2+^ sensitivity [12]. In addition, contractile kinetics were measured as the rate of torque development (RTD) at the 100 ms timepoint of each 50 Hz tetanus, which fits within the early phase suggested to capture peak force development [26]. Torque onset (i.e., 0 ms) was set using the second pulse during 50 Hz tetanus, with RTD being calculated at 100 ms (torque at 100 ms—torque at 0 ms)/0.1 s. In addition, 50 Hz half relaxation time (HRT) was measured [27], which was determined as the time from the last pulse of the 50 Hz tetanus to the force returning to 50%.

### 2.5 Intramuscular Temperature

Before inserting the intramuscular probe, the muscle belly of the tibialis anterior was located at the halfway point of the lower left leg and moving laterally from the tibia. Upon cleansing the skin with 70% isopropyl alcohol, an autoclaved tissue implantable thermocouple microprobe (Model IT‐21; Physitemp, Clifton, NJ, USA) was inserted ~2 cm into the muscle using a 21‐gauge catheter. Once inserted, the microprobe plug was connected to a temperature recorder (Model BAT‐12; Physitemp, Clifton, NJ, USA), providing intramuscular temperature measurements. To ensure the microprobe stayed in place with minimal movement, medical tape and athletic pro‐wrap were tightly secured around the microprobe insertion site to ensure the wire stayed at the depth at which it was inserted initially. Intramuscular temperature was recorded following each set of HIIE, each minute during the 10 min recovery interventions, and every 3 min for up to 3 h in the post recovery intervention period.

### 2.6 Fatiguing Exercise

Fatiguing exercise involved left‐legged repeated all‐out isokinetic concentric contractions of the dorsiflexor and plantarflexor muscles set at 160°/s, an angular velocity allowing for two contractions per second (40° of dorsiflexion +40° of plantarflexion = 80° per one full shortening and lengthening phase). The interval nature of the exercise involved six sets of 30 s all‐out contractions with 3 min of rest between sets. This exercise protocol was selected as it showed that six sets of 30 s all‐out cycling markedly increased skeletal muscle fatigue and also induced PLFFD [14]. During the exercise, participants received verbal encouragement from the experimenter and visual feedback of their dorsiflexion and plantarflexion torque and joint range of motion on a computer monitor. Immediately following the final set, neuromuscular assessments (i.e., MVC, %VA, 10 Hz, and 50 Hz) were performed to determine the extent of fatigue caused by the initial bout of exercise. The second HIIE bout, performed 24 h later, was identical to the first HIIE bout. For each set within each HIIE bout, the total number of contractions was counted. For each dorsiflexion contraction performed, peak velocity and torque were measured. Peak velocity was measured because, in the latter sets of the HIIE protocol, participants were unable to achieve 160°/s for the entire 30 s due to the onset of fatigue development.

### 2.7 Post‐Exercise Recovery Protocol

Immediately following exercise, participants underwent a post‐exercise recovery period. Before immersing the exercised leg in cold water, the lower left leg was placed in a bag to prevent direct skin contact and potential discomfort from the cold water. The CWI condition involved complete immersion of the lower left leg (up to the tibial tuberosity) in cold water and crushed ice at 10°C for 10 min. Water temperature was monitored using a digital thermometer, and crushed ice was added as needed to maintain the water temperature at 10°C. For the RT condition, participants were asked to rest seated at room temperature for 10 min in the same position that CWI was applied.

### 2.8 Statistical Analysis

Two‐way repeated measures ANOVA, a mixed‐effects model for repeated measures, and paired t‐tests were performed as appropriate using GraphPad software (*Prism* 10.2.1, San Diego, CA, USA). Firstly, all data were tested for normality using a D'Agostino & Pearson test. If data passed normality, a two‐way repeated measures ANOVA was then performed to assess the effects of the recovery interventions (CWI vs. RT) and time on changes in MVC torque (%), %VA, 10 Hz (N·m), 50 Hz (N·m), 10:50 Hz ratio, RTD (N·m/s), and intramuscular temperature (°C). In the case of HRT, initial normality testing failed, therefore outliers were identified and removed using the ROUT (*Q* = 0.5%) method, with a D'Agostino & Pearson test then being performed again to test for normality. Once the second normality test passed, a mixed‐effects model for repeated measures was then performed to assess the effects of the recovery interventions (CWI vs. RT) and time on changes in HRT (s). For the post hoc analysis, a Tukey's test was performed where appropriate. Paired t‐tests were performed to compare baseline measures of the two interventions. In addition, paired t‐tests were performed to compare performance (i.e., number of contractions, peak velocity, and peak torque achieved per set) between the two groups for the initial bout and next‐day bout of HIIE. Data within the text, figures, and tables are presented as means ± SD. Statistically significant differences were determined at *p* < 0.05.

## 3 Results

### 3.1 Baseline Measurements

There were no significant differences in baseline variables between the two experimental visits (see Table 1).

**TABLE 1:** Baseline comparison of maximal voluntary contraction (MVC) torque (N·m), voluntary activation (%), 10 Hz torque (N·m), 50 Hz torque (N·m), 10:50 Hz torque ratio, intramuscular temperature (°C), 50 Hz rate of torque development (RTD) (N·m/s), and 50 Hz half relaxation time (HRT) (s). No significance was seen between the two groups for any measures as shown in the *t*‐test *p*‐values. Data in table are presented as mean ± SD.

	RT	CWI	*p*
MVC torque (N·m)	34.8 ± 8.4	34.4 ± 10.0	0.80
Voluntary activation (%)	98.5 ± 1.0	99.2 ± 1.0	0.10
10 Hz torque (N·m)	12.5 ± 3.7	12.3 ± 3.2	0.66
50 Hz torque (N·m)	21.0 ± 7.2	19.7 ± 6.4	0.23
10:50 Hz torque	0.63 ± 0.14	0.66 ± 0.19	0.38
Intramuscular temperature (°C)	33.5 ± 0.9	33.7 ± 0.9	0.41
50 Hz RTD (N·m/s)	128 ± 45	127 ± 41	0.82
50 Hz HRT (s)	0.15 ± 0.04	0.18 ± 0.07	0.14

### 3.2 Initial HIIE Bout

**FIGURE 2:**
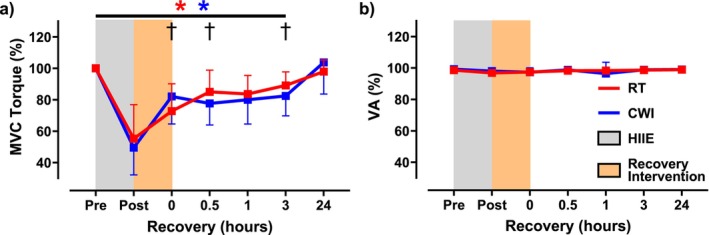
Maximal voluntary contraction (MVC) torque (%) and voluntary activation (%VA) assessed at pre and immediately following high‐intensity interval exercise (HIIE), the recovery intervention, and throughout a 24‐h recovery period beginning at 0 h. (a) MVC torque was impaired for up to 3 h in both groups compared to pre‐exercise (*p* < 0.05), with full recovery 24 h later. Significant differences between the two groups were seen at 0 h, 0.5 h, and 3 h (*p* < 0.05) (b) %VA was not impaired following HIIE in either group with no further differences seen in either group following the recovery intervention (*p* > 0.05). The red asterisk (*) signifies significant reductions in torque in the RT group from pre and the blue asterisk (*) denotes significant reductions in torque compared to pre in the CWI group.

**FIGURE 3:**
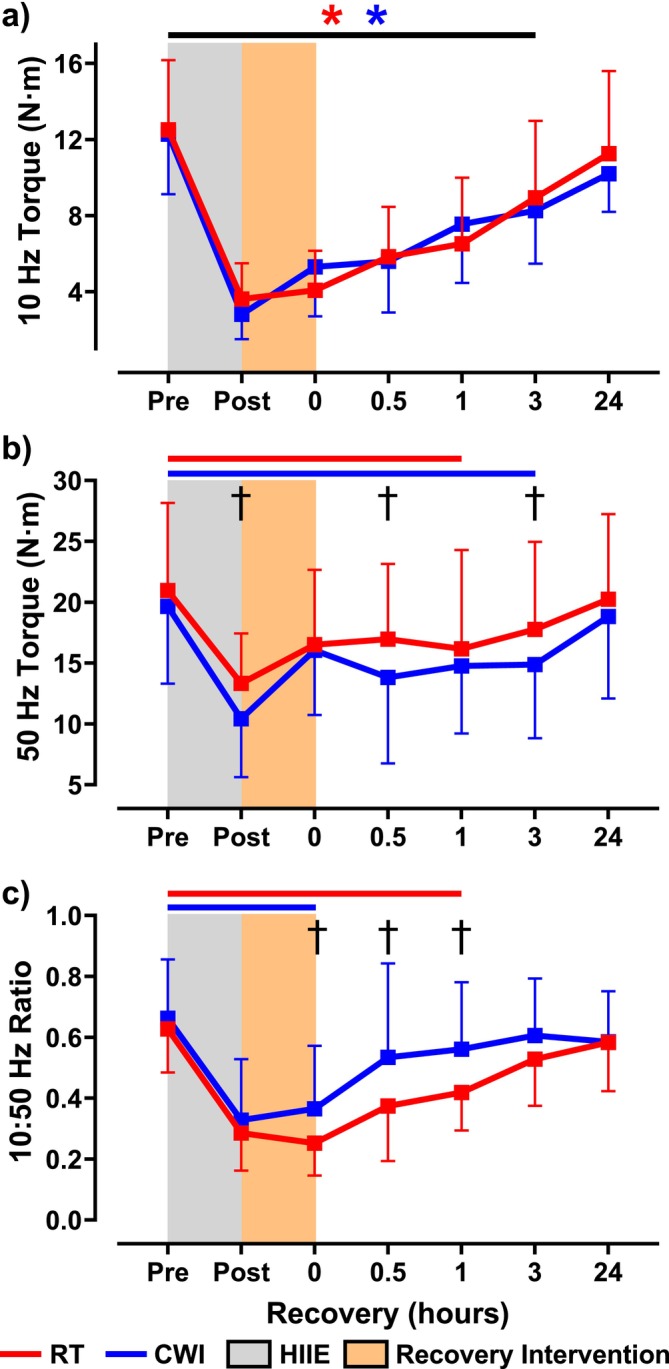
10:50 Hz torque ratio, 10 Hz torque (N·m), and 50 Hz torque (N·m) assessed at pre and immediately following high‐intensity interval exercise (HIIE), the recovery intervention, and throughout a 24‐h recovery period beginning at 0 h. (a) 10 Hz torque remained impaired for up to 3 h in both conditions (*p* < 0.05) with full recovery 24 h later. (b) 50 Hz torque remained impaired for up to 3 h following CWI compared to RT, which was impaired for up to 1 h. Significant differences between RT and CWI were seen immediately following HIIE, 0.5 h, and 3 h (*p* < 0.05). (c) 10:50 Hz torque ratio recovered immediately following CWI, whereas it remained impaired for up to 1 h following RT. Significant differences in the 10:50 Hz torque ratio were seen at 0 h, 0.5 h, and 1 h (*p* < 0.05). The red asterisk (*) and line (**―**) signifies significant reductions in torque in the RT group from pre and the blue asterisk (*) and line (**―**) denotes significant reductions in torque compared to pre in the CWI group.

**FIGURE 4:**
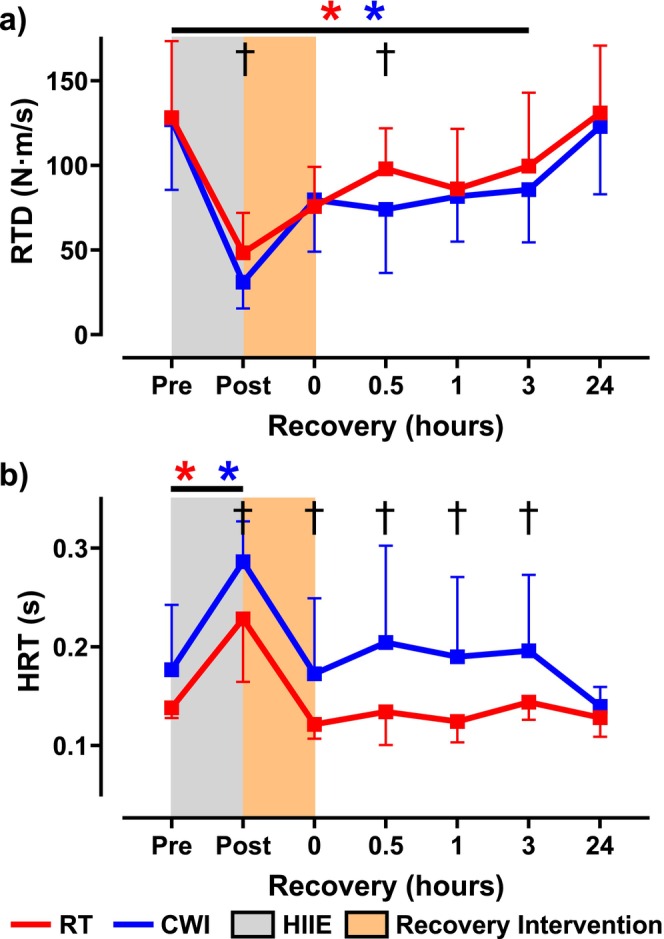
50 Hz rate of torque development (RTD) (N·m/s) and 50 Hz half relaxation time (HRT) (s) assessed at pre and immediately following high‐intensity interval exercise (HIIE), the recovery intervention, and throughout a 24‐h recovery period beginning at 0 h. (a) RTD remained impaired for up to 3 h in both conditions (*p* < 0.05). Significant differences between the two conditions were seen at following HIIE and at 0.5 h (*p* < 0.05). (b) HRT was impaired in both conditions following exercise with full recovery in both conditions following RT or CWI (0 h). However, significant differences between the two conditions were seen following HIIE, 0 h, 0.5 h, 1 h, and at 3 h (*p* < 0.05). The red asterisk (*) signifies significant reductions in torque or time in the RT group from pre and the blue asterisk (*) denotes significant reductions in torque or time compared to pre in the CWI group.

**FIGURE 5:**
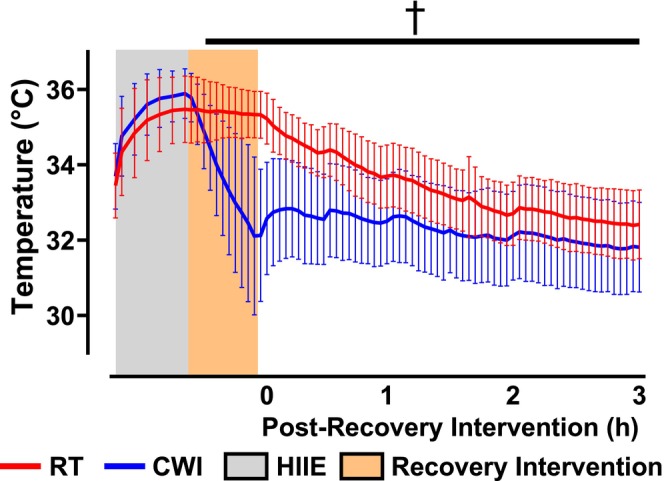
Intramuscular temperature assessed at pre, during, and immediately following high‐intensity interval exercise (HIIE), the recovery intervention, and throughout the first 3 h of the recovery period beginning at 0 h. Intramuscular temperature was assessed during HIIE following every interval. During the recovery intervention, intramuscular temperature was recorded every minute for the 10‐min period. Finally, during the recovery period, intramuscular temperature was recorded every 3 min for 3 h. Intramuscular temperature was significantly different between conditions beginning 2 min into the recovery intervention and lasting the entire 3 h recovery period (*p* < 0.05). The (†) signifies significant differences in intramuscular temperature between the two conditions.

Neuromuscular function was assessed immediately after HIIE. MVC torque significantly decreased in both conditions (RT: 55.2% ± 26.1%; CWI: 49.5% ± 17.3%) with no significant difference between them (*p* = 0.08) (Figure 2a). Voluntary activation remained unchanged within and between conditions (RT: 96.9% ± 1.0%; CWI: 98.0% ± 2.1%; *p* = 0.28) (Figure 2b). Electrically evoked contractions showed a significant reduction in 10 Hz torque (RT: 3.6 ± 1.9 N·m; CWI: 2.8 ± 1.3 N·m) with no difference between conditions (*p* = 0.22) (Figure 3a). Similarly, 50 Hz torque decreased in both conditions (RT: 13.3 ± 4.1 N·m; CWI: 10.4 ± 4.8 N·m), with a significant difference between them (*p* = 0.01) (Figure 3b). The 10:50 Hz ratio also declined (RT: 0.29 ± 0.12; CWI: 0.33 ± 0.20) without intergroup differences (*p* = 0.31) (Figure 3c). RTD was significantly reduced in both conditions (RT: 48.4 ± 23.6 N·m/s; CWI: 31.0 ± 15.5 N·m/s), with a significant difference between them (*p* = 0.04) (Figure 4a). The 50 Hz HRT increased significantly in both conditions (RT: 0.22 ± 0.06 s; CWI: 0.34 ± 0.13 s), with a significant difference between them (*p* = 0.04) (Figure 4b). Intramuscular temperature increased significantly in both conditions (RT: 35.6°C ± 0.9°C; CWI: 36.0°C ± 0.7°C), with no significant difference between them (*p* = 0.07) (Figure 5).

### 3.3 Neuromuscular Function Following the Recovery Interventions

**FIGURE 6:**
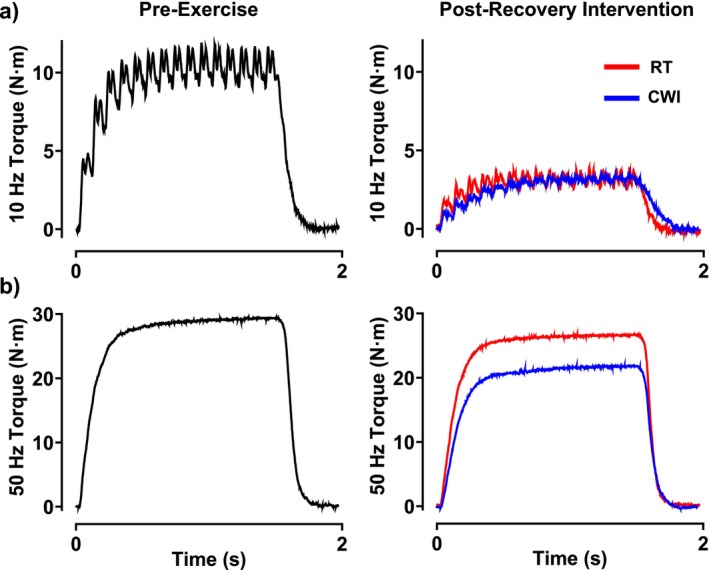
Data from a participant showing low‐ and high‐frequency force recovery following post‐exercise CWI. (a) CWI demonstrated fused 10 Hz sub‐tetanic torque resulting in similar peak torque to RT recovery. (b) CWI impaired 50 Hz tetanic torque resulting in decreased peak torque development compared to RT, which recovered faster than CWI.

When comparing the recovery of MVC torque, both interventions displayed significantly reduced MVC torque throughout the first 3 h (RT: 89.1% ± 8.6%; CWI: 82.4% ± 12.7%), which was then fully recovered 24 h later (RT: 97.9% ± 7.4%; CWI: 103.7% ± 20.1%) (Figure 2a). Significant differences following the interventions on MVC torque recovery were seen at 0 h (*p* = 0.005), 0.5 h (*p* = 0.03), and 3 h (*p* = 0.04) (Figure 2a). Similarly to the initial bout of HIIE, no significant differences were seen within or between interventions on %VA throughout the 24 h recovery period (Figure 2b). Regarding the electrically evoked contractions, 10 Hz torque remained impaired for up to 3 h following both interventions (RT: 8.9 ± 4.0 N·m; CWI: 8.3 ± 2.8 N·m), with full recovery 24 h later (RT: 11.3 ± 4.3 N·m; CWI: 10.2 ± 2.0 N·m) (Figure 3a). In addition, no differences were seen between the interventions throughout the 24 h period. Regarding 50 Hz torque, it was impaired for up to 1 h in RT (16.2 ± 8.1 N·m; *p* = 0.001), whereas following CWI, 50 Hz torque remained impaired for up to 3 h (14.9 ± 6.0 N·m; *p* = 0.001) (Figure 3b). Significant differences between the two interventions on 50 Hz torque were seen at 0.5 h (*p* = 0.007) and 3 h (*p* = 0.01) (Figure 3b). Comparing the recovery of the 10:50‐Hz torque ratio, the RT condition remained impaired for up 1 h (0.42 ± 0.12), whereas in the CWI condition, the 10:50‐Hz torque ratio recovered within 0.5 h (0.53 ± 0.31) (Figure 3c). Furthermore, significant differences were seen between the two interventions at 0 h (*p* = 0.009), 0.5 h (*p* = 0.0003), and 1 h (*p* = 0.001) (Figure 3c). When assessing RTD recovery, both interventions remained impaired for up to 3 h (RT: 99.6 ± 43.4 N·m/s; CWI: 85.7 ± 31.2 N·m/s), with full recovery at 24 h (RT: 131.0 ± 39.8 N·m/s; CWI: 123.0 ± 40.0 N·m/s) (Figure 4a). Furthermore, a significant difference between the two groups on RTD was seen at 0.5 h (*p* = 0.005) (Figure 4a). Lastly, HRT was not different within each intervention throughout the 24 h recovery period. However, significant differences between HRT were seen between the two interventions at 0 h (*p* = 0.03), 0.5 h (*p* = 0.003), 1 h (*p* = 0.005), and 3 h (*p* = 0.03) (Figure 4b). Representative data from one participant showing 10 Hz and 50 Hz force tracings are shown in Figure 6a,b.

### 3.4 Intramuscular Temperature Changes During Exercise and Recovery

Following 10 min of RT recovery immediately following HIIE, intramuscular temperature was 35.4°C ± 0.6°C, a temperature difference of −0.15°C ± 0.3°C from the beginning of RT rest. Comparatively, intramuscular temperature following 10 min of CWI recovery was 32.2°C ± 2.0°C, a temperature difference of −3.7°C ± 1.7°C from the beginning of CWI. Intramuscular temperature became significantly different between the two interventions 2 min into the post‐exercise recovery intervention (*p* = 0.02), where it remained different between RT and CWI for the remaining 3 h (*p* < 0.05) (Figure 5).

### 3.5 Next‐Day HIIE Performance

When comparing initial and next‐day performance between the two groups, no difference in the mean total number of contractions performed, peak velocity achieved, or peak torque measured (except peak torque following Set 6 during the initial bout of HIIE; *p* = 0.03) occurred between the two conditions for either bout of HIIE (Bout 1 and Bout 2) (see Table 2).

**TABLE 2:** Total contractions, peak velocity, peak torque. No significant difference was seen between either group during either bout of exercise or exercise variable except for peak torque in high‐intensity interval exercise (HIIE) Bout 1 following Set 6 (*p* < 0.05). Statistically significant differences between groups are denoted with a black asterisk (*). Data in table are presented as means ± SD.

	Total contractions		Peak velocity		Peak torque	
	RT	CWI	RT	CWI	RT	CWI
*HIIE Bout 1*						
Set 1	35.7 ± 3.2	35.6 ± 2.4	158.1 ± 3.7	156.7 ± 3.9	18.0 ± 3.4	19.2 ± 4.5
Set 2	36.0 ± 3.5	35.8 ± 2.3	155.9 ± 5.0	153.5 ± 7.8	18.1 ± 4.0	19.0 ± 4.4
Set 3	35.8 ± 3.5	36.1 ± 2.9	152.0 ± 7.7	148.0 ± 16.9	16.6 ± 3.9	18.9 ± 4.5
Set 4	35.7 ± 3.9	36.1 ± 2.5	148.2 ± 10.6	147.8 ± 16.8	16.3 ± 4.3	19.7 ± 4.6
Set 5	36.0 ± 3.3	36.0 ± 1.9	148.5 ± 11.7	146.6 ± 17.6	16.6 ± 4.7	19.3 ± 4.0
Set 6	36.0 ± 3.6	37.0 ± 2.7	148.7 ± 10.7	146.2 ± 19.9	15.8 ± 4.3*	20.2 ± 4.5*
*HIIE Bout 2 (24‐h)*						
Set 1	36.7 ± 3.7	37.3 ± 2.7	159.0 ± 3.2	158.9 ± 2.4	19.9 ± 4.2	19.1 ± 3.4
Set 2	37.0 ± 3.6	37.1 ± 2.7	158.2 ± 2.4	156.3 ± 4.8	19.2 ± 4.7	19.3 ± 3.2
Set 3	37.2 ± 3.8	36.7 ± 2.6	154.9 ± 7.8	152.2 ± 8.0	18.7 ± 5.2	18.7 ± 4.0
Set 4	36.9 ± 3.7	36.5 ± 3.0	153.3 ± 7.9	150.8 ± 8.6	17.6 ± 4.7	19.0 ± 4.4
Set 5	36.4 ± 3.2	36.5 ± 3.1	151.5 ± 8.6	149.1 ± 10.2	17.4 ± 5.0	18.4 ± 4.1
Set 6	36.1 ± 3.0	36.3 ± 2.7	148.6 ± 10.7	147.1 ± 10.9	17.5 ± 5.0	18.0 ± 4.3

## 4 Discussion

This study set out to explore the effects of cold‐water immersion following HIIE on the recovery of neuromuscular function across a 24‐h period. Furthermore, HIIE performance was assessed 24 h apart to understand the prolonged effects that CWI might have on subsequent exercise performance. Our major findings showed that CWI was neither effective in accelerating the recovery of PLFFD nor 24 h HIIE performance.

Our HIIE model performed in the ankle dorsiflexor muscles was enough to induce peripheral fatigue; however, no central fatigue was apparent, as indicated by the preservation of voluntary activation. Similarly, a previous study involving repeated Wingate exercise performed on a cycle ergometer, which involved a greater muscle mass and rise in intramuscular and core body temperature, also showed no presence of central fatigue [14]. Therefore, it is fair to suggest that fatigue induced by HIIE resided intrinsic to the muscle and contributed to the post‐exercise impairments in MVC and high‐ and low‐frequency torque observed in the current study.

We originally hypothesized that skeletal muscle function would recover quicker following CWI due to the acute recovery of the 10‐Hz submaximal torque. Mentioned earlier, previous studies have shown that following HIIE, PLFFD is present and has been shown to remain impaired for considerably longer than high‐frequency forces [13, 14] due to the presence of exercise‐induced oxidative stress. It was thought that CWI following fatiguing exercise would acutely recover low‐frequency force by reducing post‐exercise RONS generation and intramuscular temperature [4] and subsequently improving SR Ca^2+^ handling. Furthermore, by reducing intramuscular temperature, it was previously suggested that SR Ca^2+^ release would be delayed and reuptake would be more prolonged [28], thus potentially increasing myofibrillar Ca^2+^ sensitivity [29]. In particular, decreased SR Ca^2+^ reuptake might prolong relaxation time [30] between evoked contractions leading to greater force fusion with unfused tetani. In our current study, analysis of HRT showed that following CWI, HRT was significantly longer in duration compared to RT for up to 3 h into the recovery period (Figure 4b). This prolonged HRT might indicate why the low‐frequency torque tracings show a slightly more fused tracing compared to RT (Figure 6a). However, CWI did not increase peak 10 Hz torque, suggesting no temperature‐dependent effect of force fusion affecting submaximal torque. In previous studies where muscle cooling induced greater force fusion at lower stimulation frequencies, muscle temperatures were reduced to 22°C [31], which is considerably lower than what was seen in the current study where muscle temperatures immediately following CWI and RT were 32.1°C and 35.4°C, respectively (Figure 5). On the other hand, the effect of CWI seemed to have slightly delayed the recovery of high‐frequency torque (Figure 3b). One of the potential detrimental effects of post‐exercise CWI is its effects on sarcolemmal membrane excitability [4]. Specifically, CWI has been shown to prolong action potential propagation, thus resulting in delayed and slowed contraction velocity and impaired rate of force production and tension development [4, 28, 32, 33]. At high frequencies of stimulation, a prolongation of action potentials would impair the ability to propagate the subsequent action potentials when there is only a brief duration between stimuli, whereas low frequencies would have long durations between stimuli and would not be impacted by small changes in action potential depolarization and repolarization durations. In addition, it has been previously shown that when muscle temperature is reduced, maximal tetanic force is impaired [34] due to either reduced force production per cross‐bridge or a decrease in the number of cross‐bridges formed [31]. Unlike low‐frequency contractions which allow for a brief relaxation between contractions, high‐frequency contractions are already fused at a rapid rate, thereby cross‐bridge slowing will result in reduced force fusion at high frequencies. This might explain why previous studies have shown impaired power output [6, 35] and increased sprint time [5, 36] during the acute phase of recovery following CWI. In the present study, RTD was slightly reduced following CWI compared to RT and might explain the slightly lower high‐frequency torque being produced (Figure 6b), although minimal.

Regarding MVC torque, impairments were seen for both RT and CWI recovery interventions for up to 3 h (Figure 2a). Inconsistencies exist within the literature regarding MVC torque recovery following cooling conditions. A study performed in the forearm muscles showed that MVC force was reduced in cold temperatures compared to neutral or hot temperatures [37] whereas another study in the elbow flexors showed no change in MVC force under cooled, neutral, or hot temperatures [33]. It has been shown that dynamic contractions are more sensitive to temperature changes than isometric contractions [34], suggesting that CWI might not have as detrimental an effect on isometric force compared to isotonic velocity. In addition, it has been shown previously that psychological factors can have an influence on participant MVC force. Specifically, when participants performed either thermoneutral water immersion, CWI, or a thermoneutral water immersion placebo condition with a dissolvable skin cleanser that participants were led to believe was beneficial to recovery, it was both the CWI and placebo condition that displayed the greatest MVC torque recovery following HIIE [38]. Therefore, it seems that a placebo effect might exist for participants who believe that CWI is an effective recovery modality and conversely for those who do not.

When considering the effects of post‐exercise CWI on subsequent HIIE performed 24 h later, no differences were seen in either the pre‐exercise recovery of neuromuscular function or performance achieved between the two conditions during exercise. It is thought that the effects of CWI dissipated well before the second HIIE bout due to the restoration of intramuscular temperature occurring in both groups within hours of CWI the previous day, suggesting that CWI had no effect on next‐day HIIE performance (see Table 2). In contrast, previous studies have shown improved next‐day sprint repeatability [10, 39] yet impaired power output [40]. Inconsistencies in next‐day sprint exercise might be due to the selected sprint test used to assess sprint repeatability. Specifically, one study used the Loughborough Intermittent Shuttle Test to determine sprint repeatability, which is a varied exercise intensity test, and might not represent all‐out effort typically seen with HIIE [39]. Furthermore, another study used a 10‐m sprint to determine sprint performance, which is considerably shorter in duration than our HIIE model, which involved 3 min of total work (6 sets x 30‐s intervals) per HIIE bout [10]. Therefore, future studies are still needed to determine the prolonged effects that CWI might have on subsequent HIIE performance due to the varied exercise testing used to assess next‐day performance.

Some limitations exist with our study which include the absence of EMG data to determine whether prolonged action potential duration underlies the decrease in evoked 50 Hz torque following CWI as previously it has been shown that reduced temperature with cooling can impair neural conductance [4]. Another potential limitation of this study is the use of ankle dorsiflexors instead of a larger muscle group, such as the knee extensors. In using a smaller muscle group, our selected CWI protocol would be able to reduce intramuscular temperature to a greater extent than would likely be observed in larger muscle groups. Thus, we presume that longer durations of CWI may be needed to see the same effects in larger muscle groups as we observed in the current study. Another limitation of the study is the use of isometric neuromuscular assessments to assess the level of fatigue for a fatigue‐inducing protocol that involved dynamic exercise. However, our neuromuscular assessments involved electrically evoked stimulations that can only be performed isometrically. In addition, our HIIE model had a set isokinetic speed of 160°/s. However, as mentioned previously, not everyone was able to achieve that speed across the entire six sets due to the development of fatigue. Therefore, peak velocity was measured instead. Lastly, another limitation with any CWI study is the lack of participant blinding. However, with the inclusion of involuntary stimulated contractions to assess muscle function, any potential placebo effect is minimized compared to voluntary efforts.

In conclusion, CWI was not able to enhance the recovery of low‐frequency torque following HIIE compared to RT. Furthermore, there were no major acute or prolonged effects of CWI on the recovery of neuromuscular function. Lastly, CWI had no effect on next‐day HIIE performance, suggesting that post‐exercise CWI does neither improve nor impair subsequent HIIE performed 24 h later.

### 4.1 Perspective

The results of this study will provide athletes, coaches, and other performance and rehabilitation professionals information regarding post‐exercise CWI on skeletal muscle function and recovery. In this study, it was shown that CWI can slightly delay and impair the recovery of high‐frequency tetanic force, which is relevant for track and field sports where competitions can occur within hours of each other and require athletes to perform at maximal peak efforts where the difference between winning and losing can come down to milliseconds. Furthermore, we show that 10 min of 10°C CWI has no beneficial nor detrimental effect on next‐day (i.e., 24 h) neuromuscular function or exercise performance, which is relevant for team sport athletes that might have competitions on back‐to‐back nights within a span of 24 h.

## Ethics Statement

This experiment was approved by the York University Human Participants Ethics Review Committee.

## Conflicts of Interest

The authors declare no conflicts of interest.

## Data Availability

The data that support the findings of this study are available from the corresponding author upon reasonable request.
